# Evaluation of risk factors associated with the peritoneal flap hernioplasty for complex incisional hernia repair - a retrospective review of 327 cases

**DOI:** 10.1007/s10029-024-03162-1

**Published:** 2024-09-25

**Authors:** Kristian Als Nielsen, Bruce Tulloh, Andrew de Beaux, Andreas Kristian Pedersen, Sofie Ronja Petersen, Brandur Jogvansson, Mark Bremholm Ellebaek, Alexandros Valsamidis, Ayat Allah Alnabhan, Per Helligsø, Michael Festersen Nielsen

**Affiliations:** 1 Department of Surgery, University Hospital of Southern Denmark, Aabenraa, 6200 Denmark; 2 Department of Upper GI Surgery, Royal Infirmary of Edinburgh, Edinburgh, Scotland; 3 Department of Clinical Research, University Hospital of Southern, Aabenraa, Denmark; 4 Department of Surgery A, Odense University Hospital, Odense, Denmark

**Keywords:** Incisional hernia repair, Ventral hernia, Peritoneal flap hernioplasty, Complications, Risk factors, Abdominal wall reconstruction

## Abstract

**Background:**

Repair of large incisional hernias is challenging, and the risks of postoperative complications have been associated with obesity, smoking, and diabetes. The present study was conducted to determine the impact of these risk factors on short and long-term outcomes following the repair with the peritoneal flap hernioplasty (PFH).

**Methods:**

Three hundred twenty-seven patients undergoing PFH for incisional hernia repair were identified. Patient demographics and clinical data were recorded. Patients presenting signs of complications were assessed during a visit to the outpatient clinic. A multivariable regression analysis was performed to evaluate the association between BMI, smoking and diabetes, and postoperative complications.

**Results:**

The study included 157 males (48.0%) and 170 females (52.0%). Median BMI was 30.9 kg/m^2^. Diabetes was present in 13.8% of patients. 23.2% were active smokers. The recurrence rate was 2.4%. The odds ratios for postoperative complications were increased by 9% per BMI unit (*P* < 0.01), due predominantly to a rise in superficial wound infections (*P* < 0.01) and seroma production (*P* = 0.07). The adjusted odds ratio increased fourfold in patients with BMI > 40 kg/m^2^ (*P* = 0.06).

**Conclusion:**

Incisional hernia repair with the PFH technique is associated with a low risk of short and long-term complications. The risk is associated with obesity and significantly increased in patients with a BMI exceeding 40 kg/m^2^, where a fourfold increase was observed predominantly due to seroma and superficial wound infections. The recurrence rate was 2.4% and was unaltered across BMI categories. No association was established between smoking, diabetes, and the risk of all-cause complications.

## Introduction

Incisional hernias have been reported in up to 2–30% of patients undergoing laparotomy [1]. The aim of the repair is to approximate the fascial edges thereby enabling closure of the abdominal wall without undue tension.

The anterior components separation as first described by Ramirez et al. involved separating the muscular layers of the anterior abdominal wall allowing a mobilization of the rectus muscles closer to the midline thereby restoring the anatomy [2]. The procedure has subsequently been modified by Carbonell et al. by placing a large mesh in the plane between the external and internal oblique muscles [3], but onlay, sublay, intraperitoneal, and retromuscular mesh positions have also been described. These techniques have been developed based on the assumption that a successful repair requires effective mesh augmentation of the abdominal wall [4].

More recently, Novitsky et al. have introduced the posterior component separation with transversus abdominis release (TAR) [5]. This procedure is essentially a modification of the Rives-Stoppa repair with the extension of a myofascial release of the transversus abdominis muscle [6, 7]. A major benefit of this approach is that no skin flaps are raised, which has been purported to result in lower postoperative wound complications and a lower risk of recurrence [8–10].

The peritoneal flap procedure is a modification of an operation initially described by da Silva in 1971 and is an alternative to component separation [11, 12]. Several papers have reported that the PFH is associated with a low incidence of short and long-term complications [13–18]. A variety of factors may contribute to this outcome. First, by using parts of the hernial sac to bridge the fascial defect, the repair of midline hernias can in the majority of cases be conducted as a modified Rives-Stoppa repair restricting the repair to the posterior rectus sheath [14]. Similarly, when applied to the repair of transverse incisional hernias, the tissue provided by the hernial sac reduces the need for dissection in the planes of the lateral abdominal wall, which may affect the risk of complications.

Although several investigators have presented data on short and long-term outcomes following the peritoneal flap procedure, none of these have determined the effects of obesity, smoking, and diabetes on the risk of postoperative complications. The effects of these risk factors are important to determine, because differences in the dissection technique may affect the risk of complications associated with component separation and the PFH techniques [19–25].

The relationship between obesity, metabolic syndrome, and cardiopulmonary comorbidities predisposes to complications and obesity has been associated with a prolonged hospital stay, an increased risk of surgical site occurrences, and an increased risk of recurrence [21–23]. Moreover, obesity has a significant impact on postoperative morbidity and mortality, which is supported by a recent case series in which patients with obesity were demonstrated to be more prone to reoperation for recurrence than lean individuals [26]. A primary reason for this observation lies in the predisposition towards surgical site occurrences [27]. This has been demonstrated in a retrospective study of more than 900 patients in which patients with a BMI exceeding 40 kg/m^2^ undergoing laparoscopic ventral hernia repair reported an increased risk of recurrence [28]. Consequently, patients with a BMI in that range are recommended not to undergo ventral hernia repair until their weight has been significantly reduced. However, when assessing patients for abdominal wall repair the risk of the procedure must be weighed against the consequences of the physical constraints caused by overweight and the risk of incarceration of irreducible bowel segments when the repair is delayed. While these risk factors have previously been described in patients undergoing ventral hernia repair [26–28], the present study was undertaken to determine the effects of these risk factors on postoperative complications following incisional hernia repair with the PFH technique.

## Materials and methods

### Medical records

The present study is an uncontrolled case series based on data from two independent institutions. Surgical records from 368 patients undergoing incisional hernia repair were reviewed. 249 patients from the Department of Upper GI Surgery, Royal Infirmary of Edinburgh, Scotland (procedure performed between January 1st, 2010 and December 31st, 2014) and 78 patients from the Department of Surgery, Viborg General Hospital (procedure performed between November 27th, 2017 until September 3rd, 2020) fulfilled the inclusion criteria and were enrolled in the study. Patients undergoing repair of both midline and transverse incisional hernias were included. All procedures were performed by consultant surgeons specialized in hernia surgery (BT, ADB, MFN). Patients undergoing repairs using a technique other than the peritoneal flap hernioplasty were excluded, as were patients admitted for an emergency repair. This resulted in a total of 327 patients included for study.

Data was retrieved from the Lothian Surgical Audit (LSA) system, at the Royal Infirmary of Edinburgh and through the electronic file system (EPJ), the Department of Surgery, Viborg General Hospital. Viborg, Denmark. Patient notes were reviewed in February 2023.

Patient characteristics including age and sex of the patients, body mass index (BMI), operative details including defect size and mesh size, length of hospital stay, and postoperative progress at 3 months were recorded from each patient’s case notes. Data concerning preoperative abdominal CT scans, length of hospital stay, mesh size, and time of follow-up were likewise recorded. Postoperatively, the recorded cases were reviewed and assessed for short and long-term complications. Those with symptoms of complications were either telephoned or clinically reviewed by a surgical consultant or a specialist registrar. Imagining i.e. CT or US was performed when needed to document the presence or absence of complications. Patients revealing signs of recurrence defined as a protrusion of the contents of the abdominal cavity through a defect in the abdominal wall at the site of the previous repair were referred for assessment and follow-up. A CT scan was performed to confirm or refute the presence of recurrence.

Patients who smoked tobacco within one month of surgery were considered to be active smokers. Recurrent hernia was defined as a contour abnormality associated with a fascial defect detected by physical examination and/or CT scan. Hernia defect size is measured transversely in the midline hernias and craniocaudally in the transverse hernias. Chronic pain was defined as pain persisting for at least 3 months after surgery.

### Statistical analyses

**Fig. 1:**
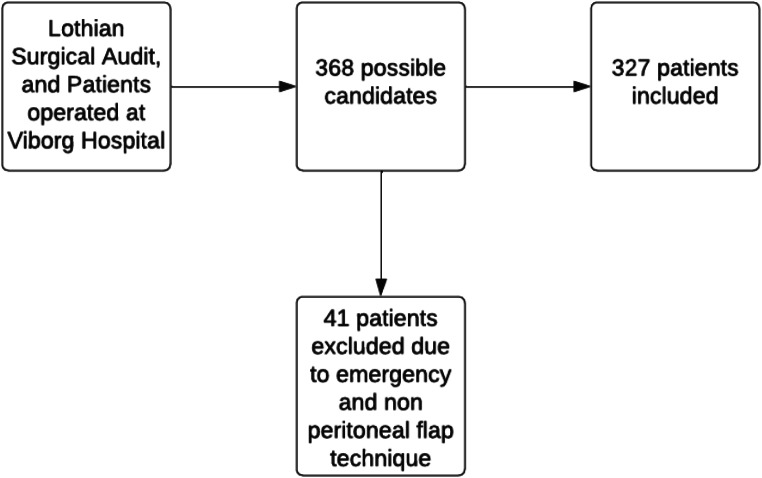
Flowchart for the inclusion of patients

To assess the reproducibility of our sample we employed descriptive statistics. Categorical variables were presented with numbers and percentages and Chi-square or Fischer’s exact tests were conducted to determine whether the distribution of categorial variables differed between the different BMI categories. The choice of test was based on Cochran’s rule. Non-categorical variables were either presented with mean and standard deviation or median or interquartile range, depending on the normality of the given variable. Likewise, for the categorial variables we assessed whether the distribution of variables differed between the BMI groups by way of statistical tests, which were either based on the Wilcoxon rank sum test or an unpaired t-test, again depending on the normality of the variables. Likewise, for the numerical variables we assessed whether the distribution of variables differed between the countries by way of a Wilcoxon rank sum test or an unpaired test, again depending on the normality of the variables (Fig. 1).

**Fig. 2:**
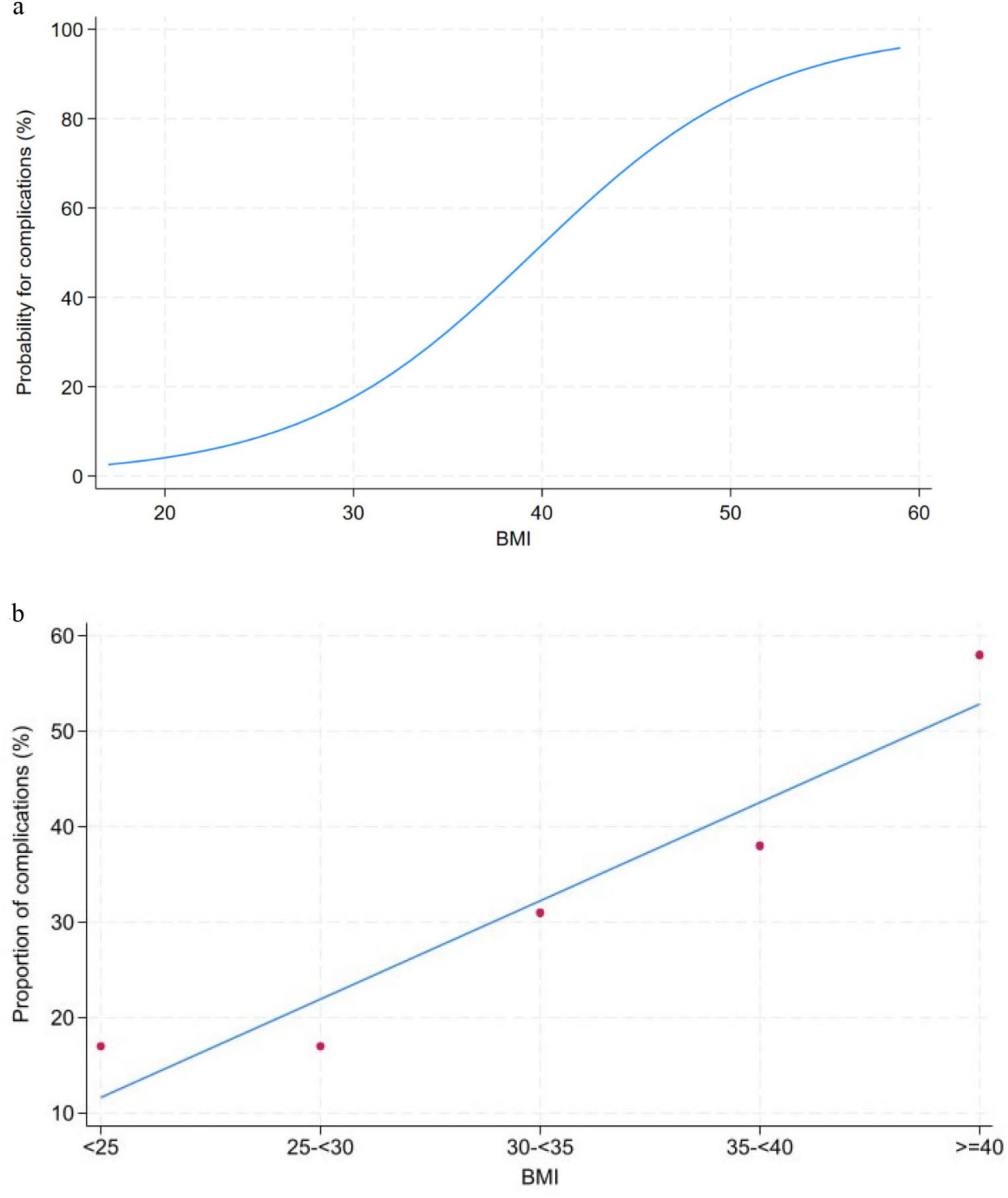
**a** Relationship between BMI as a continuous variable and the risk of all-cause complications created as a margins plot based on the logistic regression analysis. **b** Association between BMI categories and all-cause complications

The primary analysis focusing on complications was a logistic regression including BMI as exposure and adjusting for age, sex, defect size, smoking, and diabetes. To graphically depict the relation between BMI as a continuous variable and the risk of complications we created a margin plot based on the logistic regression. The model consisted of a goodness-of-fit test and a graphical assessment of linearity between the slope parameter and BMI (Fig. 2a).

**Fig. 3:**
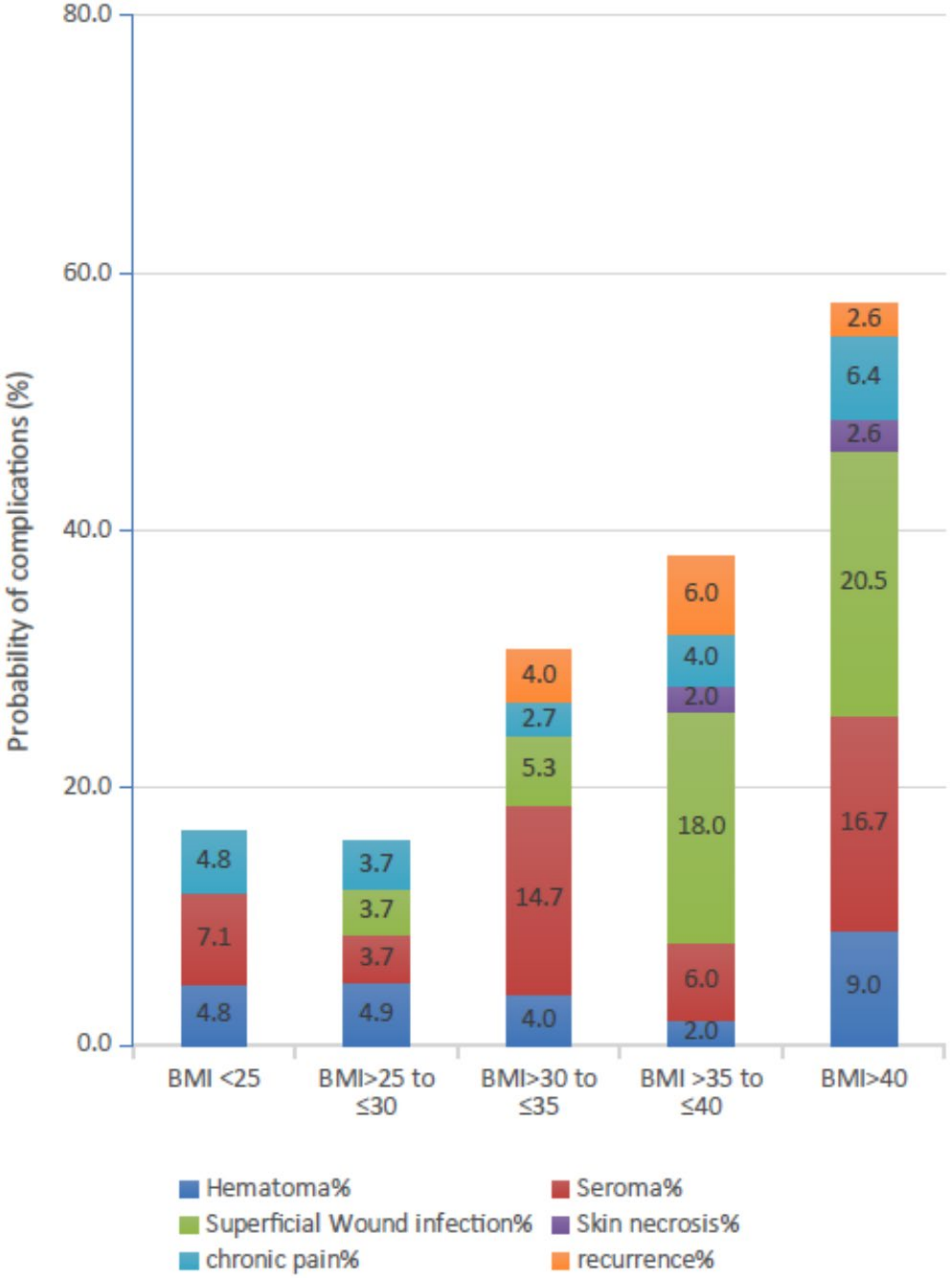
Contribution of postoperative risk factors to all-cause complications across BMI categories

Besides the primary analysis, we also conducted several secondary analyses. In the first secondary analysis, we categorized the study subjects into BMI less than 25, between 25 and 30, 30 and 35 and 40, and above 40 kg/m^2^. Again, we used logistic regression to estimate the odds ratio for each BMI group. Secondly, we conducted descriptive statistics to assess the relationship between the BMI groups and each type of complication as depicted in Figs. 2b and 3. The analysis was conducted in STATA version 18 and 905% confidence intervals and two-sided p-values were reported for all analyses. The alpha level was set to 5%.

## Results

### Patients

Three hundred twenty-seven patients were included in the study (Fig. 1). Patient characteristics from each of the two institutions are depicted in Table 1. The group consisted of 157 males (48.0%) and 170 females (52.0%). The median (IQR- interquartile range) BMI was 30.9 (27.1–36.4 kg/m2). Diabetes was present in 45 patients (13.8%), and 76 (23.2%) were active smokers at the time of surgery. Except for the median mesh size which was larger in the UK than in the Danish patients (800 vs. 375 cm^2^; *P* < 0.01), hernia location and the proportion of patients undergoing abdominoplasty, which was higher in the UK than in the Danish cohort, patient characteristics, and operative findings did not differ between the two groups (Table 1).

### Complications

The short and long-term complications for the 327 patients included in the study are listed in Table 2. Median hospital stay was 5 days (range 4–7 days). Median follow-up time was 34.5 months (range 26–41 months) for Danish patients and 114 months (range 99–127 months) for UK patients. The most common complication was superficial wound infections affecting 32 patients (9.8%). Seroma was reported in 34 patients (10.4%), while 17 patients (5.2%) experienced hematoma. Skin necrosis occurred in 3 patients (0.9%).

**Table 1:** Patient characteristics and operative findings

Factor	Level	DK	UK	*p*-value	Pooled
N		78	249		327
Age, mean ± SD		58.0 ± 13.2	59.6 ± 12.4	0.34	59.2 ± 12.6
Sex, n,%	Female	39 (50.0%)	131(52.6%)	0.70	170 (52.0%)
	Male	39 (50.0%)	118(47.4%)		157 (48.0%)
Abdominoplasty, n,%	No	53 (67.9%)	107 (43.0%)	< 0.001	160 (48.9%)
	Yes	25 (32.1%)	142 (57.0%)		167 (51.1%)
Hernia site, n%	Transverse	39 (50.0%)	83 (33.3%)	0.011	122 (37.31%)
	Midline	39 (50.0%)	166 (66.7%)		205 (62.69%)
Defect size (cm), median (IQR)		8 (5–10)	8.4 (6.2–11.4)	0.11	8 (6-10.8)
Mesh size, cm^2^, median (IQR)		375 (270–500)	800 (500–900)	< 0.001	668 (450–900)
BMI, median (IQR)		31.1 (27.5–35.6)	30.7 (27.1–36.5)	0.86	30.9 (27.1, 36.4)
Smoker, n,%	No	64 (82.1%)	187 (75.1%)	0.22	251 (76.8%)
	Yes	14 (17.9%)	62 (24.9%)		76 (23.2%)
Diabetic, n, %	No	69 (88.5%)	213 (85.5%)	0.58	282 (86.2%)
	Yes	9 (11.5%)	36 (14.5%)		45 (13.8%)

**Table 2:** Postoperative complications

Factor	
**Short term (<90 days from index surgery)**	327
Length of stay (days), median (IQR)	5 (4-7)
Hematoma, n, %	17 (5.2%)
Seroma, n, %	34 (10.4%)
Superficial wound infection, n, %	32 (9.8%)
Skin necrosis, n, %	3 (0.9%)
**Long term (> 90 days from index surgery)**	
Chronic pain, n, %	14 (4.3%)
Recurrence, n, %	8 (2.4%)
Follow up, median (IQR) in months DK	34.5 (26- 41)
Follow up, median (IQR) in months UK	114 (99-127)

There were no cases of deep mesh infection. Superficial wound infections were treated with antibiotics, local drainage and/or surgical debridement and followed in the outpatient clinic. All cases had healed within 6 months of surgery. Seromas were treated with aspiration in the outpatient clinic, some requiring several aspirations until they resolved.

Chronic pain defined as pain persisting for at least 3 months after surgery was recorded in 14 (4.3%) of patients. Skin necrosis was seen in three patients (0.9%) and one of these was extensive enough to involve the underlying fascia and peritoneal flaps, leading to mesh exposure. The wound was debrided, and vacuum dressings were applied until the patient was able to undergo revisional surgery.

Of the 249 UK patients included in the study, 40 patients had died at the time of follow-up. Thirty-seven of these causes are unrelated to the abdominal wall reconstruction. Two patients died before discharge, one from a stroke and one from myocardial infarction. One patient died from sepsis of unknown cause. 8 patients (2.4%) were identified as having a hernia recurrence. All eight had undergone surgery for recurrence by the time of closure of the study.

### Risk factors

The Odds ratios across risk factors for any complication are depicted in Table 3. A multivariate regression analysis based on these variables demonstrated an association between BMI and all-cause complications of 9% per BMI unit (1.09 range 1.04–1.15; *P* < 0.01), whereas no differences in odds of complications were established when adjusting for age, sex, hernial defect size, smoking, and diabetes (Table 3). The risk of all-cause complications as a function of BMI was calculated and depicted in a margins plot highlighting a linear relationship between BMI and the risk of complications after adjusting for potential confounders (Fig. 2a). In addition, the risk of all-cause complications was calculated for each BMI category (Table 4) and depicted in Fig. 2b showing a significant increase in the risk of all-cause complications across BMI categories (*P* < 0.01). When examining the odds ratio of complications, the adjusted risk of complications among patients with BMI exceeding 40 kg/m^2^ was demonstrated to be almost four-fold greater than the risk observed in lean individuals with a BMI less than 25 kg/m^2^ (3.98 range 0.92–17.13; *P* = 0.06) (Table 4). As depicted in Table 5 this was caused mainly by an increase in superficial wound infections (*P* < 0.01) and seroma (*P* = 0.07), whereas the risk of recurrence was demonstrated to be unaltered across the BMI categories. No association was demonstrated for skin necrosis, chronic pain, and hematoma as summarized in Fig. 3.

The sensitivity analyses concerning effect modification showed heterogeneity between the results according to geographic region with a weaker association between BMI and complications in the Danish sample than in the UK sample. No association could be established between the risk of complications and the presence or absence of an abdominoplasty across the BMI categories.

**Table 3:** Odds ratios and 95% confidence intervals across risk factors for postoperative complications

Variable	OR (95% CI)	*P*-value
BMI	1.09(1.04–1.15)	< 0.01
Age	1(0.97–1.02)	0.79
Sex	0.76(0.37–1.58)	0.47
Defect size	1.07(0.99–1.15)	0.1
Smoker	1.03(0.56–1.9)	0.93
Diabetes	0.66 (0.26–1.66)	0.37

**Table 4:** Odds ratios and 95% confidence intervals for postoperative complications across BMI categories

Variable	OR (95% CI)	*p*-value
BMI < 25	1 (reference)	
BMI > 25 to ≤ 30	0.77 (0.18–3.33)	0.73
BMI > 30 to ≤ 35	1.53 (0.46–5.14)	0.49
BMI > 35 to ≤ 40	1.69 (0.39–7.2)	0.48
BMI > 40	3.98 (0.92–17.13)	0.06

**Table 5:** Univariate associations between BMI categories and postoperative complications

BMI group	BMI < 25	> 25 to ≤ 30	> 30 to ≤ 35	> 35 to ≤ 40	> 40	*p*-value
N	42	82	75	50	78	
Recurrence	0 (0%)	0 (0%)	3 (4%)	3 (6%)	2 (3%)	0.11
Skin necrosis	0 (0%)	0 (0%)	0 (0%)	1 (2%)	2 (3%)	0.26
Superficiel wound infection	0 (0%)	3 (4%)	4 (5%)	9 (18%)	16 (21%)	< 0.001
Seroma	3 (7%)	4 (5%)	11 (15%)	3 (6%)	13 (17%)	0.07
Chronic pain	2 (5%)	3 (4%)	2 (3%)	2 (4%)	5 (6%)	0.85
Haematoma	2 (5%)	4 (5%)	3 (4%)	1 (2%)	7 (9%)	0.47

## Discussion

The PFH can be used for the repair of midline and transverse incisional hernias [14, 17, 18]. The procedure is an alternative to component separation utilizing redundant tissue derived from the hernial sac to bridge the fascial gap with mesh augmentation, allowing the hernial defect to be closed with minimal tension. While the incidence of postoperative complications has been reported in several studies [14–16] it remains unresolved to what extent obesity, smoking, and diabetes contribute to the risk profile associated with the PFH technique. This is important to determine because patients with large abdominal wall defects often struggle with the consequences of obesity.

Several studies have demonstrated that complications after laparoscopic and open ventral hernia repair increase with increasing BMI with the greatest risk observed in patients with BMI exceeding 40 kg/m^2^ [26, 29]. This observation is in accordance with the results presented in the present study demonstrating a fourfold increase in the risk of complications in morbidly obese subjects compared to lean individuals with a BMI less than 25 kg/m^2^.

Weight loss before surgery is generally recommended to minimize the risk of wound infections and hernia recurrence. However, despite vigorous efforts, attempts to achieve a stable and significant weight loss sufficient to decrease the risk of postoperative complications often fail. Mandatory preoperative weight loss may therefore at best lead to a delay in the surgical procedure with unclear advantages. Furthermore, the efficacy of preoperative weight loss on modifying postoperative outcome measures remains unclear. Preoperative weight loss is perceived to result in a reduced risk of hernia recurrence, however, to our knowledge, no studies have been able to demonstrate such an effect. Moreover, the BMI threshold at which the risk of obesity-related complications outweighs the risks associated with the repair of abdominal wall defects is not clearly defined [26, 30, 31].

Our study demonstrates a close association between BMI and the risk of all-cause complications. However, when calculating the odds ratios for BMI categories and comparing those to the risk of all-cause complications, the analysis demonstrates a significant increase in the incidence of complications among morbidly obese individuals. It is noteworthy that this increase was not due to a rise in the risk of recurrences across BMI categories, which remained virtually unaltered, but to the rise in seroma and surgical site occurrences. This outcome implies that the risk of recurrence due to being overweight may be less than hitherto assumed and that the decision to perform a repair to a greater extent should be tailored to individual patients. The presence of hernia-related discomfort and the risk of bowel strangulation and intestinal ischemia are important clinical findings that should be carefully considered, and the presence of these findings ought to weigh in on the decision of whether to perform a repair procedure in patients with obesity. The present study implies that a PFH with abdominoplasty is a safe procedure that may be considered in obese patients without the need for extensive preoperative weight loss.

Hyperglycemia has profound effects on wound healing through both immunomodulatory and vasculopathic mechanisms. Due to a blunted inflammatory response, patients with diabetes are at risk of delayed wound healing, and tight glycemic control plays a critical role in the ability of these patients to heal and fight postoperative infections [24]. Furthermore, the incidence of postoperative complications is further exacerbated by smoking [20]. Tobacco is a known risk factor for perioperative complications, in particular in obese subjects where smoking is associated with impaired wound healing [32]. The constellation of the various detrimental effects of smoking is purported to be responsible for a predisposition toward wound ischemia and infection leading to increased morbidity and mortality following ventral hernia repair [19].

In addition, smoking has been demonstrated to induce postoperative respiratory complications including pulmonary infections as well as an increased risk of surgical site occurrences, which is a direct predictor of recurrence following hernia repair. In light of these well-established adverse effects, it is noteworthy that the multivariable analyses performed in the present study did not demonstrate an association between smoking and diabetes and the presence of postoperative complications.

Several reasons may account for this outcome. First, only 8 recurrences were observed among the 327 operations (2.4%) performed. Although the present study offers no basis to conclude on this finding, a plausible explanation may be sought in the presence of too few outcome events (recurrences) among the study participants resulting in insufficient power in our analysis to demonstrate an association between these risk factors and the risk of complications. Secondly, patients with diabetes are in Denmark and the UK rigorously treated in general and before surgery, and emphasis is placed on maintaining tight glycemic control. Furthermore, the recommendations for cessation of smoking for at least 4 weeks before and 6 weeks following surgery may further have contributed to the low risk of complications associated with smoking observed in this study.

The present study has important strengths due to the large number of study participants and long follow-up, but also shortcomings and limitations, which need to be addressed. Whereas an association could be established between BMI and the risk of all-cause complications, this was not possible for smoking and diabetes. Although we have invested great efforts to ensure a correct registration of smoking habits and the presence or absence of diabetes it is impossible to rule out that misclassifications have occurred. If present this may be explained by the fact that the recordings were performed years before this information was retrieved for the study. Such a mistake would likely constitute a source of error that would weaken the precision of the multivariate analysis, particularly for diabetes and smoking habits. Furthermore, it is impossible to rule out that a certain number of surgical site events have not been recorded as some particular smaller events are likely to have been treated by the local GP. Consequently, these complications are likely not to have been entered in the patient files resulting in a potential source of error in the risk assessment.

Besides the limitation related to the sample size, the sensitivity analysis yielded effect modification between the surgical sites. The heterogeneity between the sites can either be explained by the heterogeneity between the surgeons, as one surgeon has a more stable performance than several surgeons, or by differences in patient care procedures between the two departments. Hence multicenter studies, similar to this study and contrary to previous studies [23, 24] are needed to further strengthen the reproducibility of the association between BMI and complications.

## Conclusion

The present study outlines the risk of short and long-term complications and the results from a multivariate analysis evaluating the association between BMI, smoking, diabetes, defect size, sex, and age in patients undergoing incisional hernia repair with the peritoneal flap hernioplasty. The study demonstrates that PFH is associated with a low risk of short and long-term complications and a very low risk of recurrence (2.4%). The study demonstrates that following incisional hernia repair the risk of complications is increased by BMI, in particular in patients with a BMI exceeding 40 kg/m^2^, where a fourfold increase in the risk of complications was observed. This was predominantly due to an increase in surgical site events and seroma but not to an increase in the risk of recurrence since this risk was unaltered across BMI categories in the obese subjects. No association was established between diabetes and smoking habits and the presence or absence of complications.
